# Factors associated with contemporary fatherhood

**DOI:** 10.3389/fpsyg.2024.1403955

**Published:** 2024-07-26

**Authors:** Adi Hershkovitz-Freudenthal, Osnat Lavenda

**Affiliations:** School of Social Work, Ariel University, Ariel, Israel

**Keywords:** child-centrism, intensive parenting, self-efficacy, marital satisfaction, social support, temperament

## Abstract

**Introduction:**

The most prevalent conceptualization of parenting of our time is intensive parenting which refers to parents’ overinvolvement in children’s lives, placing the child’s needs before others’ needs, including the needs of the parents themselves (i.e., Child-centrism). Intensive parenting is mostly attributed to mothers as they are still bearing the bulk responsibility for child rearing. Nevertheless, as the role of fathers changed in recent decades it is crucial to examine intensive parenting among fathers and understand whether factors that are associated with intensive mothering are associated with intensive fatherhood as well. The current study uses Belsky’s Process of Parenting model to fill-in the gap.

**Methods:**

Participants were 301 Israeli fathers of preschool children aged 22 to 50 years old (M=36.34, SD=5.01). They filled out online self-report questionnaires dealing with intensive parenting style, child temperament, social support, marital satisfaction, and parental self-efficacy.

**Results:**

The model explained 64% of paternal child-centrism. Fathers who reported having children with more difficult temperament, reported low social support, low marital satisfaction, and low self-efficacy, were more intensive in their parenting style.

**Discussion:**

The present findings are discussed in relation to previous findings regarding maternal child-centrism with an emphasis on their important implications for professionals working with families for the benefit of parents’ and children’s wellbeing.

## Introduction

In the last two decades, parents face the challenge of balancing between parental demands (i.e., investments and efforts put into the parenting role) and parental rewards (i.e., the achievement of parental goals) in order to meet the social imperatives for parenting (Nomaguchi and Milkie, 2020). The advanced technology of the current era adds more strains on contemporary parents since children are exposed to information on almost any topic one can think of. Such exposure reduces children’s need of their parents as a source of knowledge and leads to a reversed socialization process in which children teach, impact, and change parents’ views, instead of the other way around (Benedetto and Ingrassia, 2021). Such rapid social changes, which characterize Western cultures in particular, force an adjustment in the conceptualization of the parenting role (Way et al., 2013; Bjorklund and Myers, 2019). Fathers no longer stick to the traditional role of “the provider” alone but also emphasize the importance of the home and take significant part in caring for their children (Barbeta-Vinas and Cano, 2017; Gruson-Wood et al., 2022). The use of the term “new” father in the professional literature (Devault et al., 2015; Banchefsky and Park, 2016) indicates a change in the identity and declared role of fathers as well (Lamb, 2000; Cabrera et al., 2018). Fathers have become full partners as parents, from feeding and nurturing infants (Wall and Arnold, 2007) to participating in prenatal courses and the delivery process itself (Parke, 1996; Gruson-Wood et al., 2022).

Most studies show that the involvement and support of fathers in their child’s life contribute to the child’s adaptation and his/her cognitive -, motor -, emotional -, and social-development (Marsiglio et al., 2000; Cabrera et al., 2007; Lamb, 2010; Devault et al., 2015). Nonetheless, there is a lacuna in the literature regarding contemporary parenting style among fathers, particularly regarding intensive parenting, which is mostly attributed to mothers (Ishizuka, 2019), and regarding factors that contribute to the formation of this paternal style. The present study seeks to fill in this gap in knowledge.

In the past couple of decades, children are taking a center stage in the family, due to the precedence that society gives to children’s needs (Hays, 1996; Wall, 2010; Ishizuka, 2019). It appears that more than ever, parents in Western cultures that emphasize individualism and self-expression (Selin, 2014), support unconditional giving, which often compels parents to prefer their child’s needs over others’ needs, including their own (Ashton-James et al., 2013). Therefore, contemporary parents put many efforts into meeting the needs of their child, making them the center of their universe. Hays (1996) referred to this specific parenting style with the term “intensive mothering,” namely, a child-centered style (Hays, 1996; Vincent et al., 2004). In her study, Hays (1996) interviewed mothers who stated that by sacrificing their own needs and meeting their child’s needs first, they can promote optimal outcomes for their child. In another study, mothers indicated they believe that intensive mothering is the ideology that separates “good” from “bad” mothers (Guendouzi, 2005). According to the “good mother” model, mothers should be fully invested in their child’s care: physically, emotionally, psychologically, and intellectually (Wall, 2010; Birchley, 2016). Ehrensaft (1997) already postulated two decades ago that the generation of parents is both the most self-centered and child-centered generation, emphasizing the central importance of the parental role for the self-realization of the individual. Namely, parents emphasize their own happiness and tend to be fully invested in being “good” parents to their children. Putting children’s needs before self needs defines parenting style termed “child-centrism” (Ashton-James et al., 2013; Liss et al., 2013; Rizzo et al., 2013; Schiffrin et al., 2015), which thus far was studied among mothers alone (Gauthier et al., 2021). Most of the studies conducted thus far on intensive mothering refer to its impact on mothers’ mental state (see, e.g., Ashton-James et al., 2013; Kestler-Peleg and Lavenda, 2018). A handful of studies that tested the implications of this parenting style on children and the mother–child relationship show both positive and negative results (Ashton-James et al., 2013; Lavenda and Kestler-Peleg, 2018; Egami, 2024).

The examination of parental characteristics that are associated with intensive-parenting, particularly child-centrism which is a central characteristic of this parenting style (Gauthier et al., 2021), reveals its relations to maternal anxiety, defensiveness, and poor social support (Kestler-Peleg and Lavenda, 2018; Lavenda and Kestler-Peleg, 2018). Although there are several studies that have examined the associations between child-centrism and parental characteristics among mothers (Ashton-James et al., 2013; Rizzo et al., 2013; Kestler-Peleg and Lavenda, 2018), to the best of our knowledge, thus far no study has examined these associations among fathers, especially contemporary fathers. Therefore, the present study focuses on paternal characteristics and their associations with child-centric fathering. The examined characteristics are drawn from Belsky’s process of parenting model (Belsky, 1984), which is the most widely used model in the professional literature to explain the contribution of varied factors to the formation of parenting practices (Taraban and Shaw, 2018). Although the model was developed four decades ago it is still relevant to contemporary parenting. Based on Bronfenbrenner’s ecological model (Bronfenbrenner, 1979) that describes the various contexts that shape human development, Belsky (1984) developed a family-system model that includes the parent, the child, and the context in which their relationship occurs. This model lies on the understanding that families are complex systems comprising of network of relationships—complementing, interfacing, and sometimes competing (Hofferth, 2003; Whitchurch and Constantine, 2009). Belsky (1984) identified three central factors that impact parental practices: (a) the parent’s personal characteristics and resources; (b) the child’s personal characteristics; and (c) support systems, including the spousal sub-system, the workplace sub-system, and the extended social sub-system. Over the years the model has been expanded in terms of the components included under these three main factors (see, e.g., Taraban and Shaw, 2018; Fagan and Kaufman, 2022), allowing for a more nuanced and context-specific understanding of parenting practices.

The present study focuses on the examination of the factors representing Belsky’s model: a parent’s characteristic (i.e., parental self-efficacy), a child’s characteristic (i.e., child’s temperament), and context characteristics (i.e., perceived social support and marital satisfaction). Specifically, this study examines the impact of these factors on contemporary parenting, namely child-centric parenting (Ashton-James et al., 2013; Liss et al., 2013; Rizzo et al., 2013; Schiffrin et al., 2015) among fathers. In the following paragraphs we will detail the professional literature on each factor and its association with child-centrism.

Self-efficacy, a term that was coined by Bandura in the late 70s (Bandura, 1977), refers to one’s self-confidence in their ability to execute the courses of actions in the aim of producing a given attainment. It does not refer to the quality of the action or outcomes but to the mere persistence in the performance (Rodgers et al., 2014). Taken to the parenting context, parental self-efficacy refers to individuals’ perceptions of their ability to function as parents and influence their children’s development, behavior, and wellbeing (Sanders and Woolley, 2005; Gilmore and Cuskelly, 2009).

The professional literature emphasizes the importance of parental self-efficacy for the wellbeing of children; Parents who perceive themselves efficacious assist their children’s adjustment and their optimal development (Coleman and Karraker, 2003; Salonen et al., 2009). According to Belsky’s (1984) model parental self-efficacy is one of the most important factors impacting the performance of the parent. Studies conducted among fathers with high self-efficacy (Sanderson and Thompson, 2002; Jones and Prinz, 2005) revealed that these fathers tend to take greater part in raising their children, which increases their reports of satisfaction from parenting (Hudson et al., 2003) and as a result, increases their affection towards their children (Day and Lamb, 2003).

Nevertheless, the importance of parental self-efficacy for the parents themselves has been scarcely studied. In one of the few studies that examined parental self-efficacy and the practice of child-centrism among mothers, a negative association was found, indicating that mothers with high parental self-efficacy tended to be less child-centric (Lavenda and Kestler-Peleg, 2017). According to Kestler-Peleg and Lavenda (2018), child-centric mothers reported high levels of anxiety and defensiveness, which are associated with low self-efficacy (Trisnaningati, 2021). Moreover, the findings regarding gender differences in the perception of self-efficacy are inconsistent. Some studies indicate greater self-efficacy among mothers compared to fathers (Gilmore and Cuskelly, 2009), while others show no differences (Johnston and Mash, 1989; Rogers and Matthews, 2004). To the best of our knowledge, these associations were never studied among fathers.

Like in any other social interaction, the individual’s behavior depends not only on the self but on the other partner as well. Therefore, parental behavior is dependent on the child’s characteristics (Bryan and Dix, 2009). This statement stems from Belsky’s (1984) model as well as from many studies that examined the association between child temperament and parental behavior (Rothbart et al., 2006; Williford et al., 2007; Kiff et al., 2011).

The child’s temperament is evident from birth when children show marked variability in responsiveness to the environment. These responses and the mechanisms that regulate them constitute the child’s temperament (Rothbart, 2007). Temperament can be defined as individual differences resulting from complex genetic and environmental processes (Mullineaux et al., 2009). It is characterized by emotional, physical, and attentive responsiveness (Putnam et al., 2002; Saudino, 2005). Studies indicate that fathers and mothers differ in the way they respond to the child’s temperament (Van IJzendoorn and Wolff, 1997; Paulussen-Hoogeboom et al., 2007). For example, Zeanah et al. (1986) found that during pregnancy and up to 6 months of age, mothers perceived the child’s temperament as stable in three aspects: activity, rhythmicity, and moodiness. Fathers, on the other hand, perceived the child’s temperament as stable at this age-range only in activity. A longitudinal study that examined the association between parental stress and the temperament of 2- to 5-years-old children found that the higher the child’s negative responsiveness, the higher the stress experienced by the parent, which in turn affects the parent’s behavior (Williford et al., 2007). In addition, fathers of children with difficult temperament chose to spend less parent–child quality time and vice versa, fathers of children with easy temperament tended to spend more quality time with their children (Brown et al., 2011). Although child’s temperament was not examined in relation to the specific parental style of child-centrism, it appears that the child’s temperament affects parenting directly but also indirectly, through context characteristics, such as marital satisfaction (Mehall et al., 2009) and social support (Fan et al., 2020).

Marital satisfaction is an important contributor to the parent–child relationship and to the function of the family (Doherty et al., 1998; Kwok et al., 2013, 2015). Due to vagueness of its definition, the term is also known in the literature as marriage quality and/or marital adjustment (Harper et al., 2000). Although some studies refer to marital satisfaction as an external characteristic of the couple relationship, namely a contextual factor that impacts marriage life in the micro- and macro-system (e.g., life stressors, transitions, and economic factors) (Bradbury et al., 2000; Helms et al., 2014), the present study refers to it through the lens of Belsky’s (1984) parenting model, which defines marital satisfaction as the quality of the marital relationship.

The findings regarding gender differences are controversial. Some studies found no gender differences in the reports of marital satisfaction (Doss et al., 2009; Wong and Goodwin, 2009) while others indicate that men report higher marital satisfaction compared to women (Dillaway and Broman, 2001; Alqashan, 2008). Still, the quality of the marital relationship has been proven to be a central factor impacting parenting for both men and women (Belsky, 1984; Hartley et al., 2011). Fathers who reported high marital satisfaction showed high involvement in their interactions with their child (Frosch et al., 2000) that was relatively long and was characterized with warmth and emotional support during play activity (Lee and Doherty, 2007). In addition, satisfied fathers held more positive attitudes towards the parenting role and towards parent–child relationships (Kwok et al., 2013). It appears that these associations are bidirectional as fathers who were more involved in raising their children reported more marital satisfaction and so did their spouses (Dillaway and Broman, 2001; Kwok et al., 2015).

Although the association between marital satisfaction and child-centrism has not been studied yet, the association between life satisfaction and child-centrism was indicated in a study in which mothers who reported lack of life satisfaction felt compelled to prioritize their children’s needs and arrange their lives around their children (Rizzo et al., 2013). Additionally, compensatory behavior was found to characterize parents who were over-involved in their children’s lives (Vincent et al., 1980; Brody et al., 1986). Apparently, there is an association between marital satisfaction and the parental role but there is still much to reveal about this association in terms of contemporary parenting, i.e., child-centrism, and in the context of fatherhood.

As any other social role, parenting occurs in a context; it is influenced by various factors that are external to the nuclear family, e.g., social networks and interpersonal relationships (Han et al., 2015; Lavenda and Kestler-Peleg, 2018). At the transition to parenthood, social support was found to improve parental skills (Rautio, 2013), and according to Belsky (1984) total support, i.e., support from friends, family members, and spouse, increases the wellbeing of parents and their functioning. As indicated by Gameiro et al. (2011), the quality of parental care is associated with parents’ perception of the support they receive from family and friends. Furthermore, several studies (Hashima and Amato, 1994; Rodgers, 1998) found associations between poor social support, parental lack of knowledge regarding child rearing, and increased parental negative behavior towards the child. In regard to child-centrism, a study conducted by Lavenda and Kestler-Peleg (2018) showed that mothers who reported more of this parenting style reported lower levels of social support.

The reliance on social networks takes its toll on mothers as these networks set standards for the ideal “good mother” (Ishizuka, 2019). This ideal impacts mothers to adopt intensive and defensive parenting style and put their child’s needs at the center of attention on the expanse of their own wellbeing, while ignoring the heavy costs of such approach (Guendouzi, 2005; Ashton-James et al., 2013; Pedersen, 2016; Kestler-Peleg and Lavenda, 2018). It might be that like mothers, contemporary fathers use social networks to mutually share and learn from others how to raise children in an attempt to become “good fathers” (Fletcher and Stgeorge, 2011). The present study aims to answer this question by examining Belsky’s model among Israeli fathers.

To the best of our knowledge this study is the first to examine child-centrism among fathers. Most of the literature on parenting is based on studies conducted among mothers and from the perspective of child development, namely, the contribution of mothering to child development. The present study tests the three factors of Belsky’s model: (a) parent characteristics (i.e., parental self-efficacy), (b) child characteristics (i.e., child temperament), and (c) contextual characteristics (i.e., marital satisfaction and social support), and their contribution to predict child-centrism among fathers. Since the focus of the study is the agent of parenting, namely the father, the contribution of the father’s personal characteristic is examined above and beyond the child’s and context’s characteristics.

The present study is based on data collected from fathers of 3- to 6-years-old children in Israel. This age range was selected since its main developmental goal is the practice of separation-individuation (Mahler et al., 2018), in which great importance is referred to emotional availability of the parent. Children at this age range begin to develop their autonomy and therefore need the attendance and supervision of their caregiver along with the opportunities to experience in their surroundings (Bee and Boyd, 2002). These circumstances are optimal for examining the parental style of child-centrism.

Finally, the present study was conducted in Israel, a Western pro-natalist society that emphasizes the importance of the family and the social imperative to give birth (Kestler-Peleg and Lavenda, 2018). As such, Israeli society places parenting high in the hierarchy of social roles in adults’ lives, which sets the stage for child-centric parenting (Lavenda, 2021).

According to the literature review we have hypothesized that the three components of Belsky’s model will be significantly associated with fathers’ child-centrism. More specifically, since a child’s difficult temperament is known to be associated with low paternal engagement, we hypothesized that child’s difficult temperament will be negatively associated with fathers’ child-centrism. Since parental self-efficacy was negatively associated in previous studies with maternal child-centrism, we hypothesized that it will also be negatively associated with paternal child-centrism. Regarding marital satisfaction and social support, the findings in the literature were nonpersistent and sometimes controversial. Therefore, the examination of the associations between these factors and father’s child-centrism was exploratory.

## Materials and methods

### Participants and procedure

The inclusion criteria for participating in the present study were to be a male above 18 years old and to have at least one child at the age range of 3–6 years old. Participants were recruited through nonprobability snowball sampling. Following the approval of the ethic committee at the authors’ university, a link to an online survey was disseminated through social media platforms (Facebook, WhatsApp, online forums dealing with parenting and fatherhood etc.). The participants were directed to a website where they were informed about the aim of the survey, the issues they will be asked about, and the anonymous nature of the survey. They were then asked to sign an informed consent form, before proceeding to the online questionnaire. Participation was voluntary and no reward was offered.

The sample included 301 Israeli fathers between the ages of 22–50 (M = 36.34, SD = 5.01). As indicated in Table 1, participants reported a mean of 14.11 years of education (SD = 2.44) and a mean of 7.22 years in a committed relationship (SD = 3.53). Above half of the sample (56.5%) reported high socioeconomic status and a mean of two children per household (SD = 0.90). A *post hoc* power analysis was conducted to examine the strength of the analysis based on the sample size, using the G*Power3.1.9.7 software. The sample size of 301 was used for the statistical power analyses and a 9-predictor variable equation was used as a baseline. The recommended effect sizes used for this assessment were as follows: small (*f 2* = 0.02), medium (*f 2* = 0.15), and large (*f 2* = 0.35) (see Cohen, 1977). The alpha level used for this analysis was *p* < 0.05. The *post hoc* analyses revealed the statistical power for this study was 0.33 for detecting a small effect, whereas the power exceeded 0.99 for the detection of a moderate to large effect size. Thus, there was more than adequate power (i.e., power * 0.80) at the moderate to large effect size level, but less than adequate statistical power at the small effect size level.

**Table 1:** Descriptive statistics for sample’s characteristics (*N* = 301).

Variable	Mean	SD	Frequencies (%)
Age	36.3	5.0	
#Years with partner	7.2	3.5	
# Children	2	0.9	
Age of elder child	5.2	2.6	
Education (years)	14.1	2.4	
**Employment**			
Full time			66.2
Part time			28.1
Not employed			5.6
**Economic status**			
Not good			8.7
Pretty good			65.5
Very good			34.8
**Marital status**			
Committed relationship			98.4
Divorced			1.6

### Measures

In addition to the background information regarding age, education, socio-economic status, number of children and their ages, participants were asked to fill-in self-report questionnaires dealing with the study’s variables: Child-centric parenting style, parental self-efficacy, child temperament, marital satisfaction, and parental social support. All the measures that were used in the present study are well known in the literature, with proven validity and reliability:

#### Child-centrism scale

A 7-item scale developed by Ashton-James et al. (2013) to measure the extent to which parents prioritize their children’s needs (e.g., *“the needs of my children come before my own”; “My schedule revolves around my children”*). Participants were asked to respond to each item on a 7-point Likert scale ranging from 1 (not at all) to 7 (very much). A final mean score was calculated, with a higher score reflecting greater child-centrism. Cronbach’s alpha that was calculated in the present study was 0.95.

#### Parenting sense of competence questionnaire—PSOC

A 17-item scale developed by Johnston and Mash (1989) to measure parents’ sense of success in fulfilling the parental role (e.g., *“Being a parent is manageable and any problems are easily solved”; “Being a parent makes me tense and anxious”*). Participants were asked to respond to each item on a 4-point Likert scale ranging from 1 (not at all) to 4 (very much). A final mean score was calculated, with a higher score reflecting greater parental self-efficacy. Cronbach’s alpha that was calculated in the present study was 0.97.

#### Colorado child temperament inventory—CCTI

A 30-item scale developed by Rowe and Plomin (1977) that comprises 6 subscales measuring children’s dimensions of personality: Sociability, emotionality, activity, attention span-persistence, reaction to food, and soothability (e.g., *“child makes friend easily”* for sociability; *“child gets upset easily”* for emotionality; *“child is very energetic”* for activity; *“play with a single toy for long periods of time”* for attention span-persistence; *“rarely looks at new food without fussing”* for reaction to food; *“child tolerates frustration well”* for soothability). Participants were asked to respond to each item on a 5-point Likert scale ranging from 1 (not at all like my child) to 5 (a lot like my child). A mean score for each subscale was calculated, as well as a final mean score for the full scale, with higher score reflecting greater temperament difficulties. Cronbach’s alpha for the subscales ranged from 0.88 to 0.93. Cronbach’s alpha for the full scale was 0.91.

#### Evaluation and nurturing relationship communication and happiness—ENRICH

Evaluation and Nurturing Relationship Communication and Happiness – ENRICH. A 15-item scale developed by Fowers and Olson (1993) to measure idealistic distortion (5 items) and marital satisfaction (10 items). The present study used only the 10 items measuring marital satisfaction (e.g., *“I am very happy with how we handle role responsibilities in our marriage”; “I am not happy about our communication and feel my partner does not understand me”*). Participants were asked to respond to each item on a 7-point Likert scale ranging from 1 (strongly disagree) to 5 (strongly agree). A final mean score was calculated, with a higher score reflecting greater marital satisfaction. Cronbach’s alpha that was calculated in the present study was 0.86.

#### Perceived social support scale

A 9-item scale developed by Westman et al. (2004) to measure the perceived support from three social agents: spouse, family, and friends. Participants are asked to refer to each of these agents regarding received support (e.g., *“To what extent do you share with your friends what have happened to you recently?”*). Participants were asked to indicate how well the items describe their relationships with their spouse/family/friends on a 5-point Likert scale ranging from 1 (not at all) to 5 (very much). A final mean score was calculated, with a higher score reflecting greater perceived support. Cronbach’s alpha for the three subscales, i.e., support from spouse, support from family, and support from friends, ranged from 0.88 to 0.93.

### Data analysis

To test the contribution of the characteristics of the child, the context, and the parent, a hierarchical regression analysis was conducted. Demographic characteristics of the father (i.e., age, education, economic status, number of children, and number of years with partner) were covaried to eliminate biased results due to individual differences. Therefore, demographic variables were entered in the first step of the regression, then child and context characteristics were entered and finally, parent characteristics were entered at the third step of the regression. Data analysis was conducted using IBM SPSS statistics, version 26.

## Results

Table 2 presents correlation coefficients between the study’s variables. As indicated in the table, all the correlations indicate strong and significant relations between the dependent variable, child-centric fathering, and dependant variables (i.e., child difficult temperament, marital satisfaction, social support, and paternal self-efficacy). The strongest association was found between paternal self-efficacy and paternal child-centrism (r = −0.70; *p* < 0.001), indicating that the less fathers reported parental self-efficacy, the more child-centric style was reported. Contextual characteristics (i.e., Marital satisfaction and social support) were also negatively associated with child-centrism, while child’s characteristic (i.e., difficult temperament) was positively associated with it.

**Table 2:** Correlation coefficients between the study’s variables.

	Child-centrism	Child temperament	Marital satisfaction	Social support	Self-efficacy
Child-centrism	1				
Child difficult temperament	0.28^**^	1			
Marital satisfaction	−0.55^**^	0.04	1		
Social support	−0.60^**^	−0.04	0.66^**^	1	
Self-efficacy	−0.70^**^	−0.06	0.67^**^	0.68^**^	1

**p < 0.001.

To examine the contribution of child-, contextual-, and parent-characteristics to explain child-centric fathering, a hierarchical regression analysis was conducted. Results are summarized in Table 3. The analysis revealed a model that explains 63.2% of the variance in child-centric fathering. Demographic variables explained 9% of the variance while child difficult temperament, marital satisfaction and social support added 46.4% to the explained variance. Finally, fathers’ characteristic, namely paternal self-efficacy, contributed additional 7.8% to explain the variance in child-centrism among fathers. All steps of the hierarchical regression analyses were statistically significant. The variance inflation factor (VIF) estimates ranged between 1.092 and 2.607, indicating low multicollinearity (Thompson et al., 2017).

**Table 3:** Results of hierarchical regression analysis.

Step	Variable	*B*	SE	Beta	95% CI	*R*^2^ change	*F*
1						0.090	5.72^**^
	Age	−0.02	0.03	−0.07	−0.07, 0.03		
	Education	0.06	0.04	0.08	−0.02, 0.13		
	SES	−0.65	0.16	−0.24^**^	−0.96, −0.34		
	# Children	−0.24	0.15	−0.13	−0.52, 0.05		
	# Years with partner	0.11	0.04	0.24^*^	0.03, 0.19		
2						0.464	98.81^**^
	Age	−0.03	0.02	−0.09	−0.07, 0.01		
	Education	0.07	0.03	0.11^*^	0.02, 0.13		
	SES	−0.42	0.11	−0.16^**^	−0.64, −0.20		
	# Children	−0.27	0.10	−0.15^*^	−0.47, −0.06		
	# Years with partner	0.12	0.03	0.26^**^	0.06, 0.18		
	Child difficult temperament	1.82	0.34	0.22^**^	1.15, 2.50		
	Marital satisfaction	−0.47	0.08	−0.33^**^	−0.62, −0.32		
	Social support	−0.66	0.09	−0.38^**^	−0.84, −0.48		
3						0.078	59.97^**^
	Age	−0.03	0.02	−0.08	−0.06, 0.01		
	Education	0.06	0.03	0.09^*^	0.01, 0.11		
	SES	−0.27	0.10	−0.10^*^	−0.47, −0.07		
	# Children	−0.23	0.09	−0.13^*^	−0.41, −0.04		
	# Years with partner	0.11	0.03	0.24^**^	0.06, 0.16		
	Child difficult temperament	1.67	0.31	0.20^**^	1.06, 2.28		
	Marital satisfaction	−0.23	0.08	−0.17^*^	−0.38, −0.09		
	Social support	−0.36	0.09	−0.21^**^	−0.54, −0.18		
	Paternal self-efficacy	−0.89	0.12	−0.43^**^	−1.12, −0.66		

*p < 0.05; **p < 0.01.

Among the demographic variables, number of years with partner was the strongest characteristic associated with fathers’ child-centrism (B = 0.11, SE = 0.03, β = 0.24, t = 4.14, *p* < 0.001). It appears that fathers who are in a relationship for longer periods of time report higher levels of child-centrism. Age was the weakest and only insignificant demographic variable indicating that child-centric fathering is independent of the father’s age.

Among the independent variables and as indicated by the correlations, paternal self-efficacy was found to be the strongest predictor (B = −0.89, SE = 0.12, β = −0.43, t = −7.74, *p* < 0.001) of paternal child-centrism, above and beyond all other model variables. It was negatively associated with paternal child-centrism. Marital satisfaction and social support were also negatively associated with paternal child-centrism (B = −0.23, SE = 0.08, β = −0.17, t = −3.10, *p* = 0.002; B = −0.36, SE = 0.09, β = −0.21, t = −3.89, *p* < 0.001, in accordance). Child’s difficult temperament was positively associated with paternal child-centrism (B = 1.67, SE = 0.31, β = 0.20, t = 5.36, *p* < 0.001).

## Discussion

The present study is the first to examine Belsky’s determinants of parenting model among fathers. It is also the first to examine the model in relation to intensive parenting style, which is prevalent in the current era (Kestler-Peleg and Lavenda, 2018; Gauthier et al., 2021; Lavenda, 2021). The present findings support Belsky’s (1984) model indicating the importance of all three components in predicting parenting: The characteristics of the child, the characteristics of the parenting context, and the characteristics of the parent. The examined model explained 64% of child-centric parenting style, which provides proof for the validity and solidity of the model.

Another contribution of the present findings is the examination of factors that are associated with child-centric fathering, a parenting style that was mostly examined thus far among mothers. Contrary to our hypothesis, the findings reveal that fathers who are more child-centric in their parenting style report having children with more difficult temperament. It is possible that the implications of children’s temperament, in terms of behavior problems and adjustment to everyday life demands, force their fathers to be more engaged and focused on their child’s needs and prioritize them. This is particularly true for fathers in the present era, who share more aspects of the parenting tasks with their spouses and therefore are required more often to meet their children’s various needs (Ishizuka, 2019). It is also possible that fathers who are less child-centric implicitly convey a message to their child that they must be independent and get along with less paternal involvement. Attaining this independence from the child may be experienced and interpreted by the father as a relatively easy temperament. Since our study used paternal reports of child’s temperament this should be further examined in future studies through other means.

The present findings also reveal that fathers who report more child-centrism report low social support, low marital satisfaction, and low self-efficacy. The finding regarding social support is in line with the literature on the powerful impact that social support has in terms of social capital (Lavenda and Kestler-Peleg, 2017). Social capital is associated with high self-esteem and self-worth (Han et al., 2015) which enables parents to develop other aspects of their lives, aside from their parenting role and beyond meeting their children’s needs. Presumably fathers with poor social networks and low support invest more time and efforts in their parental role and parent–child relationship, as found among mothers (Lavenda and Kestler-Peleg, 2017). In addition, greater social capital means extended social networks that indicate that these fathers integrate other social activities in their lives, other than their parent–child relationship, and therefore they are less child-centric.

The same considerations apply to the negative correlation found between marital satisfaction and child-centrism among fathers. It appears that fathers who report high marital satisfaction, like mothers, are less concerned with prioritizing the needs of their children. According to the compensation model (Kouros et al., 2014), fathers who are satisfied with their marital relationship feel less compelled to focus on their paternal role and their parent–child relationship. They feel more able to attend to other aspects of social and family life than their parental role.

Finally, the present findings reveal a negative association between fathers’ self-efficacy and child-centrism, which is also consistent with previous findings among mothers. According to Lavenda (2021), parenting is a social role that involves intensive daily stressors and demands, and is set to meet very high social standards, almost unattainable. Therefore, this role puts parents at risk for stress, anxiety, and overall low mental health. A parent with high self-efficacy reduces these conditions by having the confidence to successfully fulfill this complicated role. Consequently, parents with high self-efficacy feel less threatened by the challenges and demands of the parenting role and feel less pressured to meet or prioritize their child’s needs.

It is also possible that fathers with high self-efficacy are less dependent on social approval because they are less child-centric in their parenting. As mentioned earlier, child-centrism is a parenting style that is mostly driven by an unwritten social imperative (Hays, 1996; Liss et al., 2013; Rizzo et al., 2013). Therefore, the fact that fathers who report low child-centrism report high self-efficacy implies that they are less vulnerable to social critique.

It is noteworthy that the findings of the present study confirm the paramount importance of parental self-efficacy above and beyond the contribution of the other factors in Belsky’s (1984) model to explain parenting and particularly child-centrism among fathers. Parental self-efficacy was found to be a significant predictor of child-centrism above and beyond demographic background (i.e., age, education, economic status, number of children, and number of years with partner), beyond differences in child’s characteristics and beyond differences in contextual characteristics. Indeed, parental self-efficacy has become a major component in the field of parenting and is one of the main indicators of appropriate parental functioning and well-being (Albanese et al., 2019). Because it is an acquired skill that is developed through experience with this role and overcoming the challenges that parenting presents, the present findings highlight the ability of parents to modify and improve their performance in the parenting role. In addition, future studies should examine the potential moderating role of parental self-efficacy in the associations between contextual and child characteristics and fathering style.

In summary, both fathers and mothers who demonstrate child-centrism parenting style are parents who are fully invested in their parenting role and are therefore characterized by poorer relationships and are less developed in other aspects of their life. The marital relationship of such parents may also be affected by such a parenting style, as parents who focus entirely on their parenting role may neglect their marital system. In addition, a parent who adopts a child-centrism parenting style may focus more on their child’s difficulties, which may cause them to invest even more resources in their role as parents and experience more difficulties in coping. Such experiences could raise doubts about their ability to fulfill their parenting role. Future studies should expand the investigation of this parenting style to the context of child-level outcomes to better understand its consequences.

The findings of the present study should be interpreted cautiously due to several limitations in the study’s design and sample. First, the design of the study is cross-sectional. To better understand the relationships between the factors in the model in terms of directionality, future studies should use a longitudinal design. Although several variables that were tested in the model are known to precede father’s intensive parenting (i.e., the dependent variable), such as child’s temperament, bidirectional associations cannot be ruled out. It is recommended for future studies to use longitudinal designs to better understand the associations between the model variables. In addition, further variables should be examined that represent the three factors in Belsky’s model and were not examined in the present study. Such variables should be the parents’ own childhood experiences, anxiety, insecurities and high expectations of their child. The children’s characteristics should also be expanded. Second, the data in the present study are based on self-report questionnaires, which could have biased the findings due to social desirability (Holden and Passey, 2009). Third, data were collected through convenience sampling and therefore the sample lacks representativeness in terms of education and economic status. It is recommended to include more representative samples in future studies from different cultures, age groups and socio-economic status. And finally, the present data did not include children’s characteristics other than temperament. Parenting style changes according to children’s age, gender, birth order etc. For example, literature on gendered parenting indicates differences in parental practices based on the child’s gender (Kane, 2018) that perpetuate gender roles in multitude life domains (Morawska, 2020) and that. These variables should be covaried out to examine the model with greater accuracy.

Despite these limitations, the present study provides primary and important insight to contemporary fatherhood. The present results point to a hierarchy in the structure of the three factors comprising Belsky’s Process of Parenting model. As far as we know, such a hierarchy has not yet been investigated. Fathers’ self-efficacy appears to be a key factor in shaping their parenting role and should be investigated in the future as a mediator in the context of the other factors. The present findings have further theoretical implications for our understanding of gendered similarities in parenting roles. It appears that intensive fathering, like mothering, is prevalent among fathers with low social support, low marital satisfaction, and children with difficult temperaments. Most importantly, however, intensive fathering is associated with low self-efficacy. Professionals working with parents and children should be aware of the present findings and develop services not only for mothers but for fathers as well. These services should focus on fathers’ perceptions of their ability to fulfil the parenting role, develop fathers’ social skills for the benefit of their relationships with their spouses and social networks, and strengthen their coping strategies for the challenges associated with the parenting role. Practitioners and policymakers should be aware of the current father figure, its parenting style and the factors that shape this style. More particularly, the social imperative for parents to prioritize their children’s needs should be acknowledged and efforts should be made to increase both fathers’ and mothers’ social networks, improve marital relationships, and increase parents’ self-efficacy for the wellbeing of mothers, fathers, and their children.

## Data availability statement

The raw data supporting the conclusions of this article will be made available by the authors, without undue reservation.

## Ethics statement

The studies involving humans were approved by Ariel University Ethic Committee [approval No. AU-SOC-OL-20191030]. The studies were conducted in accordance with the local legislation and institutional requirements. The participants provided their written informed consent to participate in this study.

## Author contributions

AH-F: Conceptualization, Data curation, Project administration, Writing – original draft. OL: Conceptualization, Formal analysis, Supervision, Writing – review & editing.
